# Predictors of biologic-free disease control in patients with rheumatoid arthritis after stopping tumor necrosis factor inhibitor treatment

**DOI:** 10.1186/s41927-019-0071-x

**Published:** 2019-06-13

**Authors:** Marjan Ghiti Moghadam, Femke B. G. Lamers-Karnebeek, Harald E. Vonkeman, Peter M. ten Klooster, Janneke Tekstra, Barbara van Schaeybroeck, Ruth Klaasen, Marieke van Onna, Hein J. Bernelot Moens, Henk Visser, Annemarie M. Schilder, Marc R. Kok, Robert B. M. Landewé, Piet L. C. M. van Riel, Mart A. F. J. van de Laar, Tim L. Jansen, on behalf of the Dutch National POET Collaboration

**Affiliations:** 1 Department of Rheumatology, Medisch Spectrum Twente, Enschede, The Netherlands; 2 Department of Psychology, Health & Technology, University of Twente, PO BOX 217, 7500 AE Enschede, the Netherlands; 3 Department of Rheumatology, Radboud University Medical Center, Nijmegen, The Netherlands; 4 Department of Rheumatology, University Medical Center Utrecht, Utrecht, The Netherlands; 5 Department of Rheumatology, Albert Schweitzer Hospital, Dordrecht, The Netherlands; 6 Department of Rheumatology, Meander Medical Centre, Amersfoort, The Netherlands; 7 Department of Clinical Immunology and Rheumatology, Academic Medical Center, Amsterdam, The Netherlands; 8 Department of Rheumatology, Ziekenhuis Groep Twente, Hengelo, The Netherlands; 9 Department of Rheumatology, Rijnstate, Arnhem, The Netherlands; 10 Department of Rheumatology, Medical Centre Leeuwarden, Leeuwarden, The Netherlands; 11 Department of Rheumatology and Clinical Immunology, Maasstad Hospital, Rotterdam, The Netherlands; 12 Department of IQ Healthcare, Radboud University Medical Center, Nijmegen, The Netherlands; 13 Department of Rheumatology, VieCuri Medical Center, Venlo, The Netherlands

**Keywords:** Rheumatoid arthritis, Remission, Low disease activity, TNFi discontinuation, Predictors

## Abstract

**Background:**

The aim of this study was to identify predictors of prolonged disease control after discontinuation of tumor necrosis factor inhibitor (TNFi) treatment in patients with rheumatoid arthritis (RA).

**Methods:**

Post-hoc analysis of 439 RA patients (67.3% rheumatoid factor positive) with longstanding RA in remission or with stable low disease activity, randomized to stopping TNFi treatment in the multicenter POET trial. Prolonged acceptable disease control was defined as not restarting TNFi treatment within 12 months after stopping. Baseline demographic and disease-related variables were included in univariate and multivariate logistic regression analysis for identifying predictors of relapse.

**Results:**

One year after baseline, 220 patients (50.1%) had not restarted TNFi treatment. Use of an anti-TNF monoclonal antibody (versus a receptor antagonist, OR = 2.41; 95% CI: 1.58–3.67), ≤10 yrs. disease duration (OR = 2.15; 95% CI: 1.42–3.26) and low or moderate multi-biomarker disease activity (MBDA) scores (OR = 2.00; 95% CI: 1.10–3.64) at baseline were independently predictive of successful TNFi discontinuation (area under the receiver operating characteristic curve = 0.66; 95% CI: 0.61–0.71). Results were similar when using no physician-reported flare as the criterion. TNFi-free survival was significantly different for patient groups based on the number of predictors present, ranging from 21.4% of patients with no predictor present to 66.7% of patients with all three predictors present.

**Conclusion:**

Patients using an anti-TNF monoclonal antibody, with shorter disease duration and low or moderate baseline MBDA score are most likely to achieve prolonged disease control after TNFi discontinuation.

**Trial registration:**

Netherlands Trial Register NTR3112, 21 October 2011.

**Electronic supplementary material:**

The online version of this article (10.1186/s41927-019-0071-x) contains supplementary material, which is available to authorized users.

## Background

Treatment options and outcomes in rheumatoid arthritis (RA) have improved markedly over the last decades [1–3]. Particularly the use of conventional synthetic disease modifying anti-rheumatic drug (csDMARDs) in combination with the more expensive biological agents (bDMARDs such as tumor necrosis factor inhibitors (TNFi) has been shown to be effective in reducing disease activity, improving physical function, and slowing radiographic damage [4–6]. In practice, patients achieving low disease activity (LDA) or even remission usually continue this combination therapy permanently. This, however, may lead to unnecessary treatment and resource use, as several studies have suggested that a substantial number of RA patients in sustained remission or LDA can taper or altogether stop their TNFi without experiencing a disease flare [7, 8].

Although current guidelines suggest that tapering or withdrawal of bDMARDs can be considered for patients in persistent remission [9], where persistent remission is not explicitly defined, validated predictors of successful discontinuation are needed to successfully implement practical stopping rules in daily clinical practice [10]. A number of studies have already examined predictors of successful TNFi discontinuation, but it remains a challenge to judge which patients can effectively and safely stop their TNFi without incurring a flare of the disease [11]. A recent systematic review found no consistently strong predictors for successful dose reduction or discontinuation [12]. Only shorter disease duration [13–15], less or no erosive disease [14–16], and lower disease activity [14–17] at baseline have been shown in more than one study to predict successful TNFi discontinuation. Additionally, single studies have found that younger age [14], normal body mass index (BMI) [16], non-smoking [15], anti-cyclic citrullinated peptide antibodies (ACPA) negativity [17, 18], shorter [15] or longer [19] TNFi treatment, and initial (versus delayed) TNFi treatment [13] were associated with successful TNFi discontinuation. Finally, two studies have found a tendency for more relapses among female patients [13, 17].

Moreover, two main types of TNFi with different mechanisms of action are used in RA: anti-TNF monoclonal antibodies versus soluble TNF receptors (receptor antagonist). Monoclonal antibody agents like adalimumab and infliximab bind specifically to TNF, while the receptor fusion protein etanercept, functioning as a decoy receptor, binds to circulating TNF and prevents it from interacting with a cell surface receptor [20–23]. Adalimumab and etanercept are currently the most frequently prescribed TNFi and generally demonstrate comparable effectiveness [24, 25]. Previous discontinuation studies, however, generally examined one specific TNFi agent only or did not examine possible differences in the predictive value of the type of TNFi (anti-TNF monoclonal antibody or receptor antagonist) for successful discontinuation.

Recently, the POET trial showed that, although stopping TNFi treatment in patients with established RA in remission or with stable low disease activity resulted in substantially more flares than did continuation of TNFi, around half of the patients could successfully stop for at least 12 months [26]. The aim of the current post-hoc study was to identify baseline predictors of prolonged disease control after discontinuation of TNFi.

## Methods

### Patients and study design

We used data from the Dutch pragmatic POET trial, registered in the Netherlands Trial Register (NTR3112) [26]. This multicenter trial randomized RA patients with stable LDA, as measured with the 28-joint Disease Activity Score using the erythrocyte sedimentation rate (DAS28-ESR) [27], in a ratio of 2:1 to either discontinuing or continuing TNFi treatment. For those patients randomized to stopping TNFi, concomitant treatment with csDMARDs was continued. Eligible patients had to fulfill the ACR 1987 criteria for RA, be 18 years or older, and have received TNFi treatment for ≥1 year. Patients also needed to have stable LDA for ≥6 months before inclusion, which was operationalized as either two DAS28-ESR scores < 3.2 or the rheumatologist’s clinical judgment of remission or stable low disease activity with at least one C-reactive protein (CRP) measurement < 10 mg/L in the last 6 months. Finally, no dosage changes were allowed to have occurred for csDMARDs or corticosteroids in the 6 months prior to inclusion.

A total of 531 patients were randomized to the stop group [26]. In case of a disease flare, defined as a DAS28-ESR score ≥ 3.2 with an increase > 0.6 [28], the treating rheumatologist could re-initiate TNFi treatment. As the current study aimed to identify patient and clinical characteristics predictive of prolonged disease control after discontinuation of TNFi treatment, only the data from 439 patients randomized to the stop group and for whom baseline serum samples were collected to measure the multi-biomarker disease activity (MBDA) score [29] were used. Baseline characteristics were comparable between the included 439 patients with and the 92 patients without an available baseline MBDA measurement [30].

### Measures

Included patients were examined by the rheumatologist and rheumatology nurse at baseline and return visits were scheduled at 3, 6, 9 and 12 months or earlier if the symptoms suggested a disease flare. The following measurements were collected at baseline: age, sex, weight, height, body mass index (BMI), disease duration, rheumatoid factor (RF) and ACPA status, erosive disease (yes/no) and medication use, including the type of TNFi (antibody vs. receptor antagonist) and concomitant csDMARD use. In a substantial subsample, the MBDA score was assessed at baseline. The MBDA score is a novel test that measures 12 biomarkers in serum to assess disease activity in patients with rheumatoid arthritis [29]. The scoring algorithm generates a total score from 1 to 100, with definitions for low (< 30), moderate (30 to 44) and high (> 44) disease activity [31].

Standard disease activity measurements were collected at every visit and consisted of a 28-joint tender and swollen joint count (TJC28 and SJC28), laboratory measurement of the ESR (mm/h) and CRP (mg/l) and a patient-reported general health assessment on a 100-mm visual analog scale (VAS-GH). The TJC28, SJC28, ESR and VAS-GH were used to calculate the DAS28-ESR as a composite index of disease activity [27]. Physician-reported flares and medication changes were continuously recorded.

### Statistical analysis

Successful discontinuation was defined as not restarting TNFi treatment within 12 months after stopping. First, univariate logistic regression analyses were performed to examine associations between potential baseline predictors and successful 12-month TNFi discontinuation. The following patient and clinical variables were considered: type of TNFi (anti-TNF monoclonal antibody (adalimumab, infliximab, golimumab or certolizumab)) vs. receptor antagonist (etanercept), concomitant csDMARD use, female sex, younger age (≤60 yrs.), shorter disease duration (≤10 yrs.), RF negativity, ACPA negativity, non-erosive disease, normal weight (BMI = 18.5–25), first TNFi agent, deep remission (DAS28 ≤ 1.98) [32, 33], and low or moderate MBDA score (≤44) [30]. To facilitate the interpretation of odds ratios (ORs), continuous predictors were dichotomized by median split (DAS28-ESR, age, disease duration) or previously established relevant cut-off points (BMI, MBDA). ORs are classified as weak when about 1.5, moderate when about 2.5, strong when about 4 and very strong when about 10 [34]. Additional separate models were run to test for possible interactions between each predictor and type of TNFi. Predictors univariately associated with outcome (*P* < 0.10) were included in a multivariate logistic regression model to identify independent predictors. The multivariate model was reduced by excluding predictors from the model with *P* > 0.10 (backward deletion), and the goodness of fit of the final model was estimated using the Hosmer and Lemeshow test [35], where a non-significant result indicates support for the model. The predictive ability of the model was examined using the area under the receiver operating characteristic (ROC) curve. All regression analyses were based on observed data. As 38 patients (8.7%) had missing baseline values on laboratory and radiographic erosion data, the multivariate model was repeated using multiple imputation with 10 imputed datasets. Since results were very similar to non-imputed data, only the results from observed data are presented. As a sensitivity analysis, the final multivariate model was repeated using no physician-reported flare within 12 months after stopping as the dependent variable. To further explore the prognostic value of the remaining predictors, TNFi-free survival based on the number of predictors present was examined using Kaplan-Meier survival analysis. Between-group difference in survival was tested using the log rank test. Since MBDA testing is not common in many rheumatology practices, the multivariate and survival analyses were repeated without MBDA as a predictor. All statistical calculations were performed using SPSS 23.

## Results

Table 1 summarizes the baseline characteristics of the patients (*n* = 439). Median age and disease duration was 60 and 10 years, respectively. Approximately 60% of the patients used an antibody agent, most frequently adalimumab, while 40% used etanercept. Most patients were on their first TNFi. In total, 219 (49.9%) of the patients restarted their TNFi within 12 months, while at least one physician-reported flare was reported for 251 (57.2%) patients. Forty-four patients with a physician-reported flare (17.5%) did not restart their TNFi, while no physician-reported flare was recorded for 12 patients who restarted TNFi (6.4%).

**Table 1:** Baseline characteristics of the patients (*N* = 439)

Female, n (%)	296 (67.4%)
Age (yrs.), mean (SD)	59.8 (10.8)
Disease duration (yrs.), median (IQR)	10 (6–17)
BMI, mean (SD)	25.9 (4.3)
Normal BMI (18.5–25), n (%)	174 (39.6%)
RF positive, n (%)	270 (67.3%)
ACPA positive, n (%)	277 (69.1%)
Erosive disease, n (%)	252 (62.8%)
ESR, median (IQR)	9.0 (5–17)
TJC28, median (IQR)	0 (0–1)
SJC28, median (IQR)	0 (0–0)
PGA, median (IQR)	20.7 (9.0–28.1)
DAS28-ESR, mean (SD)	2.0 (0.8)
MBDA score, mean (SD)	30.2 (12.6)
Low (< 30) or moderate (30–44), n (%)	375 (85.4%)
Type of TNFi, n (%)	
Etanercept	176 (40.1%)
Adalimumab	225 (51.3%)
Infliximab	22 (5.0%)
Golimumab	14 (3.2%)
Certolizumab	2 (0.5%)
Number of TNFi, n (%)	
1st	379 (86.5%)
2nd	50 (11.4%)
3rd	9 (2.1%)
csDMARD, n (%)	
Methotrexate	362 (82.5%)
Methotrexate + glucocorticoids	20 (4.6%)
Glucocorticoids	6 (1.4%)
Other csDMARD	29 (6.6%)
No DMARD	22 (5.0%)

*TNFi* tumor necrosis factor-alpha inhibitors, *DAS28* disease activity score in 28 joints, *BMI* body mass index, *RF* rheumatoid factor, *ACPA* anti-cyclic citrullinated peptide antibodies, *ESR* erythrocyte sedimentation rate, *CRP* C-reactive protein, *TJC28* 28-joint tender joint count, *SJC28* 28-joint swollen joint count, *PGA* patient global assessment, *MBDA* multi-biomarker disease activity, *csDMARD* conventional synthetic disease modifying anti-rheumatic drug

Antibody type TNFi, shorter disease duration, non-erosiveness and low or moderate MBDA were weakly to moderately associated with successful discontinuation, defined as not restarting TNFi treatment within 12 months after stopping in univariate regression analysis (Table 2). No interactions with type of TNFi were significant and separate univariate analyses for both types of TNFi showed that the predictive value of individual variables was similar for patients discontinuing an antibody agent or etanercept. However, MBDA ≤44 was significantly predictive only in patients discontinuing etanercept (OR = 3.69; 95% CI: 1.34–10.18; *P* = 0.012) and not in patients discontinuing an antibody agent (OR = 1.68; 95% CI: 0.81–3.45; *P* = 0.162).

**Table 2:** Univariate associations of baseline variables with successful TNFi discontinuation

Predictor	OR	95% CI	P
anti-TNF monoclonal antibody^a^	2.26	1.53–3.34	< 0.0001
Concomitant DMARD	1.21	0.51–2.85	0.670
Female sex	1.07	0.72–1.60	0.735
Younger age (≤60 yrs.)	1.17	0.80–1.70	0.417
Shorter disease duration (≤10 yrs.)	2.00	1.34–2.98	0.001
RF negative	1.14	0.75–1.74	0.530
ACPA negative	1.06	0.70–1.62	0.775
Non-erosive	1.62	1.08–2.44	0.020
Normal weight (BMI 18.5–25)	1.35	0.92–1.98	0.128
First TNFi	1.23	0.71–2.13	0.461
DAS28 deep remission (DAS28 ≤ 1.98)	1.21	0.83–1.77	0.314
Low or moderate MBDA (≤44)	2.32	1.32–4.05	0.003

^a^Reference category is receptor antagonist

*TNFi* tumor necrosis factor-alpha inhibitors, *csDMARD* conventional synthetic disease modifying anti-rheumatic drug, *RF* rheumatoid factor, *ACPA* anti-cyclic citrullinated peptide, *BMI* body mass index, *DAS28* disease activity score in 28 joints, *MBDA* multi-biomarker disease activity

In multivariate analysis, non-erosiveness lost its significance (OR = 1.34; 95% CI: 0.85–2.11; *P* = 0.212). Type of TNFi, shorter disease duration and low or moderate MBDA scores remained independently predictive of successful discontinuation, with type of TNFi being the strongest predictor (Table 3). The area under the ROC curve showed a modest predictive ability of 0.66 for these three variables. This decreased to 0.65 when omitting MBDA, suggesting rather limited added value of MBDA as a predictor for biologic-free disease control above and beyond type of TNFi and disease duration. Logistic regressions with disease duration and MBDA as continuous predictors demonstrated very similar results, with erosiveness again losing significance and type of TNFi, shorter disease duration and lower MBDA scores remaining independently predictive of successful discontinuation [see Additional file 1].

**Table 3:** Multivariate associations with successful TNFi discontinuation with and without MBDA score as a predictor

	With MBDA			Without MBDA		
Predictor	OR	95% CI	P	OR	95% CI	P
anti-TNF monoclonal antibody^a^	2.41	1.58–3.67	< 0.0001	2.46	1.61–3.73	< 0.0001
Disease duration < 10 yrs.	2.15	1.42–3.26	< 0.0001	2.14	1.42–3.23	< 0.0001
MBDA score ≤ 44	2.00	1.10–3.64	0.023	–	–	–

^a^Reference category is receptor antagonist

*TNFi* tumor necrosis factor-alpha inhibitors, *MBDA* multi-biomarker disease activity, *OR* Odds ratio. Hosmer and Lemeshow with MBDA χ2(5) = 1.57, *P* = 0.905, area under ROC curve = 0.66 (95% CI: 0.61–0.71, *P* < 0.0001); Hosmer and Lemeshow without MBDA χ2(2) = 0.00, *P* = 1.000, area under ROC curve = 0.65 (95% CI: 0.59–0.70, *P* < 0.0001)

As a sensitivity analysis, using ‘no physician-reported flare’ as the criterion for successful TNFi discontinuation resulted in similar findings in the total sample, with the same three predictors remaining significant in multivariate analysis. However, the predictive value of the variables tended to be slightly lower with ORs of 1.86 (95% CI: 1.24–2.79; *P* = 0.003), 1.78 (95% CI: 1.18–2.70; *P* = 0.006) and 2.49 (95% CI: 1.35–4.59; P = 0.003) for antibody TNFi, shorter disease duration and low or moderate MBDA score, respectively.

**Fig. 1:**
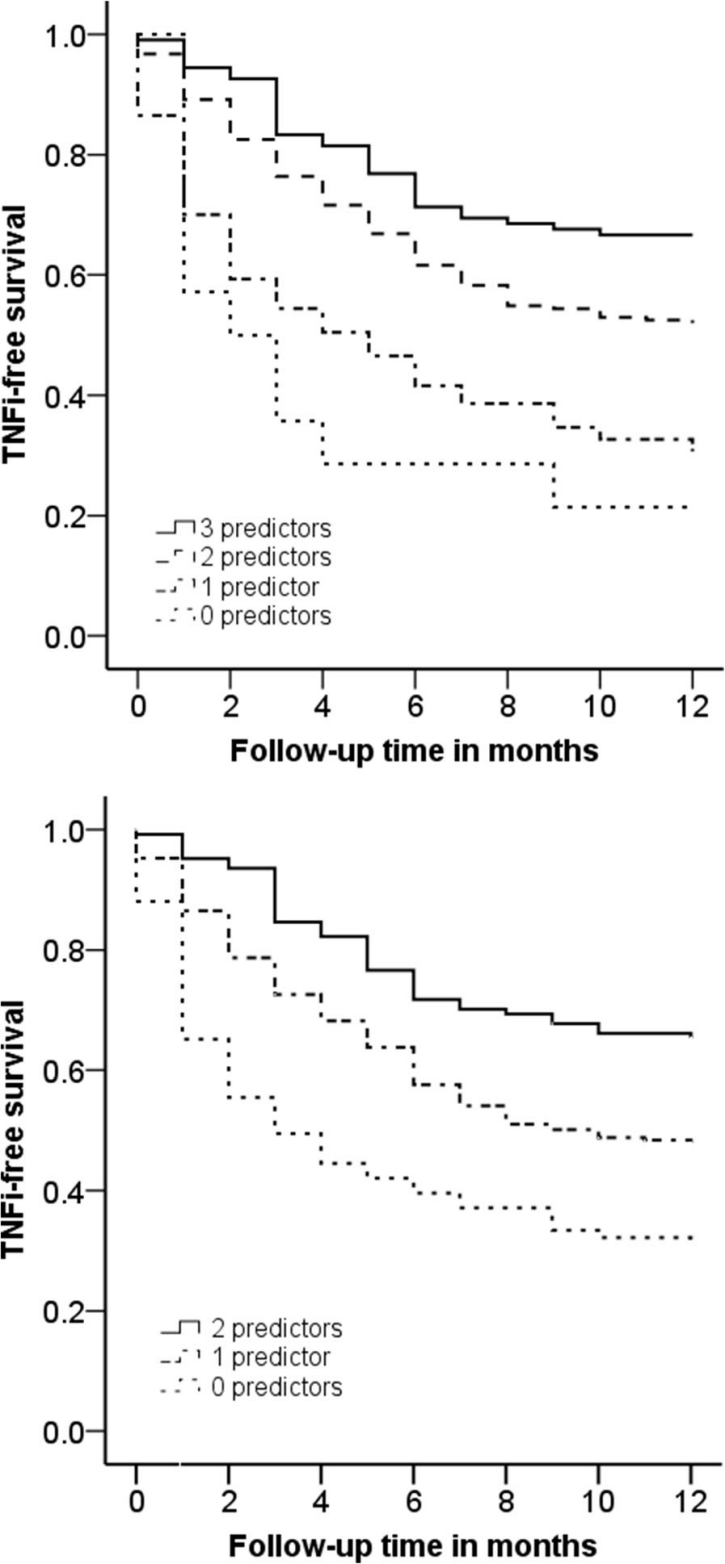
Kaplan-Meier curves showing the proportion of patients not restarting TNFi per number of predictors present. Upper panel: with MBDA as predictor; Lower panel: without MBDA as predictor. MBDA = multi-biomarker disease activity

TNFi-free survival was significantly different (log rank = 43.9, *P* < 0.001) for patient groups based on the number of predictors present (Fig. 1). TNFi-free survival rates were 21.4% in patients with no predictor present (*n* = 14), 31.7% in patients with one predictor (*n* = 104), 52.6% in patients with two predictors (*n* = 213), and 66.7% in patients with three predictors (*n* = 108) present. Fairly similar results and differences between groups (log rank = 33.9, P < 0.001) were obtained in TNFi-free survival when using only antibody type TNFi and shorter disease duration as predictors. In this analysis, TNFi-free survival rates were 32.1% in patients with no predictor present (*n* = 84), 48.5% in patients with one predictor (*n* = 231), and 65.3% in patients with both predictors (*n* = 124) present.

## Discussion

The POET study previously demonstrated that in patients with established RA in sustained remission or with stable low disease activity, as defined by the DAS28-ESR, approximately 50% of the patients could successfully stop their TNFi for at least 12 months [26]. However, with the consequential fifty-fifty chance of relapsing, it would be helpful to identify patient and clinical predictors of prolonged disease control after discontinuation of TNFi. This post-hoc analysis showed that the type of TNFi (anti-TNF monoclonal antibody vs. receptor antagonist) being used, RA disease duration and the MBDA score at the time of discontinuation of TNFi were significantly associated with success of stopping. Approximately 70% of patients who were on an anti-TNF monoclonal antibody, with a disease duration of < 10 years and a MBDA score ≤ 44 were able to successfully stop their TNFi, while conversely 80% of patients on a TNFi receptor antagonist, with a disease duration of ≥10 years and a MBDA score > 44 restarted their TNFi within 12 months of stopping.

Several other studies have previously explored potential predictors of disease relapse after TNFi discontinuation [11, 12]. Multiple studies found shorter disease duration, RF positivity, non-smoking, erosive disease and normal body mass index (BMI) to be predictive of successful TNFi discontinuation [14, 16]. In the current study, shorter disease duration was also a predictor of successful discontinuation, but RF positivity and normal BMI were not. Erosive disease was univariately associated with restarting TNFi, but did not remain a unique predictor in the final multivariate model.

Higher clinical disease activity at the time of discontinuation has also previously been identified as a predictor, although results varied considerably between studies [13, 14, 36]. In the current study, higher baseline DAS28-ESR scores were not associated with the criterion of restarting TNFi treatment within 12 months. However, a high MBDA score at the time of TNFi discontinuation was predictive of restarting TNFi. Several previous studies found that MBDA scores may be elevated when conventional clinical measures indicate remission or LDA [18, 37–39]. Furthermore, these patients were found to be at increased risk for progressive joint damage [37–39]. In patients with high baseline MBDA scores at the time of TNFi discontinuation in POET, discontinuation may have allowed a resurgence of subclinical residual inflammation and the subsequent need to restart TNFi treatment [18].

At the time of the analysis, the type of TNFi, i.e. anti-TNF monoclonal antibody vs. receptor antagonist, had not been previously identified as a predictor of successful TNFi discontinuation. Very recently, however, Hashimoto et al. also found that use of infliximab, adalimumab, and golimumab, as opposed to etanercept or certolizumab pegol, was clearly more advantageous for achieving bDMARD-free remission in a retrospective registry study of patients discontinuing bDMARDs [40]. This is in line with the current findings, which also showed that patients who were using an anti-TNF monoclonal antibody (mostly adalimumab) were significantly more often able to successfully discontinue their TNFi than patients who had been using a receptor antagonist (mostly etanercept). These findings might be explained by several differences between the TNF receptor antagonists and antibodies. Firstly, TNF receptor antagonists function as decoy receptors that bind to circulating TNF, thereby mimicking the inhibitory effects of naturally occurring soluble TNF receptors, albeit with a greatly extended circulatory half-life. TNF antibodies also bind specifically to TNF and thus block its interaction with the p55 and p75 cell surface TNF receptors. However, TNF antibodies may also lyse surface TNF expressing blood cells in the presence of complement thereby possibly inducing prolonged disease control [20–23]. Secondly, pharmacokinetic properties differ between the drugs, with a single dose half-life of approximately 4 days for the TNF receptor antagonists etanercept vs. 10–20 days for the TNF antibody adalimumab [20]. Finally, there may be differences in drug dosages and dosage-intervals.

This current post-hoc analysis used a relatively large dataset collected in the POET study, a randomized controlled trial on stopping TNFi in RA patients in daily clinical practice. The subgroup of 439 patients appeared to be an unbiased sample of the total stop group. With an average age of 60 years and a median disease duration of 10 years, the sample also appeared to be representative of the population with established RA using TNFi in The Netherlands.

However, the study also has several limitations that should be considered. Besides the general limitations of exploratory post-hoc data analyses [41], one specific limitation of the current analysis is that we only focused on baseline predictors. Longitudinal (especially early) changes in variables such as the DAS28, ESR/CRP or MBDA scores could have provided insight into the potential for monitoring of these variables to predict relapse. Another possible limitation of the POET study was the decision to leave restarting of TNFi at the discretion of the treating rheumatologists. It is possible that this has led to an overestimation of successful disease control and bias in the population of TNFi restarters. Finally, due to the pragmatic nature of the POET study, a small proportion of patients were still using prednisone at baseline, even though current guidelines clearly state that glucocorticoids should have been withdrawn before bDMARDs are tapered [9].

## Conclusions

In patients with established RA in sustained remission or with stable LDA, use of an anti-TNF monoclonal antibody, disease duration less or equal to 10 years and low to moderate MBDA score at the time of discontinuation were significantly associated with success of stopping TNFi. Although their predictive ability was only modest, it may be useful to consider these clinical characteristics when deciding to stop TNFi treatment in RA patients in daily clinical practice. The current findings and robustness of the specific predictors should be confirmed in other samples of RA patients stopping TNFi treatment. Also, future studies could focus more on the predictive value of early changes in disease-related variables and on other potential markers for successful TNFi discontinuation.

## Additional file

Additional file 1: Multivariate associations with successful TNFi discontinuation with and without MBDA score as a predictor, with disease duration and MBDA as continuous predictors. (DOCX 13 kb)

## Data Availability

The datasets generated and analyzed for the current study are not publicly available due to legal restrictions related to data privacy protection. However, the data are available upon reasonable request to all interested researchers after authorization of the POET steering committee. Researchers interested in data access may contact the PI of the POET study Tim L. Jansen (tjansen@viecuri.nl) to apply for access.

## Abbreviations

ACPA: Anti-cyclic citrullinated peptide antibodies
bDMARD: Biological disease modifying anti-rheumatic drug
BMI: Body mass index
CRP: C-reactive protein
csDMARD: Conventional synthetic disease modifying anti-rheumatic drug
DAS28: Disease activity score in 28 joints
DMARD: Disease modifying anti-rheumatic drug
ESR: Erythrocyte sedimentation rate
LDA: Low disease activity
MBDA: Multi-biomarker disease activity
OR: Odds ratio
PGA: Patient global assessment
RA: Rheumatoid arthritis
RF: Rheumatoid factor
SJC28: 28-joint swollen joint count
TJC28: 28-joint tender joint count
TNFi: Tumor necrosis factor-alpha inhibitors
