# Supplementary material for: Predictors of biologic-free disease control in patients with rheumatoid arthritis after stopping tumor necrosis factor inhibitor treatment
Source: BMC Rheumatol. 2019 Jun 13;3:3. doi: 10.1186/s41927-019-0071-x (PMC6567570; doi:10.1186/s41927-019-0071-x)
Supplement: Supplementary file 1 — Multivariate associations with successful TNFi discontinuation with and without MBDA score as a predictor, with disease duration and MBDA as continuous predictors. (DOCX 13 kb) [file 41927_2019_71_MOESM1_ESM.docx]

**Additional file 1**

Multivariate associations with successful TNFi discontinuation with and without MBDA score as a predictor, with disease duration and MBDA as continuous predictors

|  | **With MBDA** | | |  | **Without MBDA** | | |
| --- | --- | --- | --- | --- | --- | --- | --- |
| **Predictor** | **OR** | **95% CI** | **P** |  | **OR** | **95% CI** | **P** |
| Antibody TNFi^a^ | 2.41 | 1.58 – 3.68 | <0.0001 |  | 2.43 | 1.59 – 3.69 | <0.0001 |
| Disease duration | 0.95 | 0.93 – 0.98 | <0.0001 |  | 0.95 | 0.93 – 0.97 | <0.0001 |
| MBDA score | 0.98 | 0.97 – 1.00 | 0.043 |  | – | – | – |

^a^ Reference category is receptor antagonist.

TNFi = tumor necrosis factor-alpha inhibitors; MBDA = multi-biomarker disease activity; OR = Odds ratio. Hosmer and Lemeshow with MBDA χ2(8) = 2.29, P = 0.971, area under ROC curve = 0.68 (95% CI: 0.62 – 0.73, P <0.0001); without MBDA χ2(8) = 5.09, P = 0.748, area under ROC curve = 0.67 (95% CI: 0.62 – 0.72, P <0.0001).
